# Exiles: Medieval Experiences of Isolation

**DOI:** 10.1007/s11061-022-09741-w

**Published:** 2022-10-15

**Authors:** Erin Sebo

**Affiliations:** Flinders University, (English), Adelaide, (South Australia) Australia

**Keywords:** Medieval, Queenship, Exeter Book, Old English Poetry.

## Abstract

For most people alive today, the COVID-19 pandemic was our first experience of widespread isolation. However, among medieval cultures, with low population density and limited urbanisation, isolation, especially through exile, was common as a political expedient or even, as now, as a method of controlling the spread of illness. This is reflected across myriad aspects of medieval culture, from pilgrim badges to legal codes. Stories and tropes of isolation are common in medieval literature. From the *immrama* which often include depictions of the isolation of voyages, to images of homesickness in romances or Crusade narratives to descriptions of isolation in exile in Old English elegies and Old Norse sagas. In many instances, the literature reveals a greater fear of loneliness than death, so much so that isolation was used both as a form of punishment considered as severe as mutilation in some parts of medieval Europe, and as an important religious practice, since many people willingly distanced themselves from society in pursuit of salvation through hardship. This introductory essay introduces a dossier on medieval experiences of isolation.

For most people alive today, the COVID-19 pandemic was our first experience of widespread isolation. However, among medieval cultures, with low population density and limited urbanisation, isolation, especially through exile, was common as a political expedient or even, as now, as a method of controlling the spread of illness. This is reflected across myriad aspects of medieval culture, from pilgrim badges to legal codes. Stories and tropes of isolation are common in medieval literature. From the *immrama* which often include depictions of the isolation of voyages, to images of homesickness in romances or Crusade narratives to descriptions of isolation in exile in Old English elegies and Old Norse sagas. In many instances, the literature reveals a greater fear of loneliness than death, so much so that isolation was used both as a form of punishment considered as severe as mutilation in some parts of medieval Europe, and as an important religious practice, since many people willingly distanced themselves from society in pursuit of salvation through hardship.

The three articles in this dossier take very different approaches to exploring medieval experiences of, and responses to, isolation and loneliness, in their various forms, as well as the complex history of the reception of medieval literary depictions of isolation. The first, Soper’s article on *The Wanderer*, considers how this early medieval image of isolation has been mediated by modern ideas, especially that of pathetic fallacy. She argues that the poem is not, as modern scholars have often taken it to be, so much interested in a specific, individual experience of exile and loneliness but rather of these conditions as universalised spiritual problems. The next article also considers the reception of texts, but this time by a near contemporary audience. Anlezark explores the interactions between medieval exiles and their own culture’s construction of exile. Focusing on the experiences of medieval bibliophiles, Anlezark examines the phenomenon of medieval exiles who collected literary texts concerned with exile. The article charts the interactions between literary constructions and lived experiences of isolation. The collection is completed by Firth and Schilling who examine the particular kinds of exile experienced by royal women, specifically the practice of exiling queens after the death of their husbands. This article looks at the dynamics of that exile but also interrogates its implication; that it suggests the beginnings of cultural and political perceptions of English queenship as an ‘office’ with immutable legitimacy and powers. Each of these articles offers an original and methodologically innovative insight into medieval experiences that are at once very removed from our own but that, increasingly, through the pandemic, offer startling parallels.

